# Why Be Funny: The Influence of Social Norms on the Communicative Functions of Humor

**DOI:** 10.3390/bs15010015

**Published:** 2024-12-27

**Authors:** Nathan Miczo, John C. Meyer

**Affiliations:** 1 School of Communication and Media, Western Illinois University, Macomb, IL 61455, USA; 2 School of Media and Communication, The University of Southern Mississippi, Hattiesburg, MS 39406, USA; john.meyer@usm.edu

**Keywords:** social norms, functions of humor

## Abstract

Humor is a valued social activity and, as such, should be influenced by social norms. This investigation examined the relationships between the functions of humor and the theory of normative social behavior. Descriptive norms are the foundation of TNSB. However, the theory argues that those norms are influenced by a set of moderators that can strengthen or attenuate proposed relationships. One hundred and sixty-three college students completed measures of TNSB variables (descriptive norms, injunctive norms, outcome expectation, group identification, and reward) as well as Ramsey and Meyer’s functions of humor scale (identification, clarification, enforcement, and differentiation). Though descriptive norms significantly correlated with all four humor functions, in regression analyses, no significant associations emerged. On the other hand, injunctive norms consistently predicted humor functions. In moderation analyses, the interaction between descriptive norms and reward was significant for all four functions of humor. The discussion highlights the role that normative mechanisms play in shaping the ways people use humor in their everyday lives.

## 1. Introduction

In many interactions, possessing and displaying a sense of humor is a valued attribute, one aspect of which is the ability to employ humor in social interaction, through relating funny stories, repeating jokes, and “chaining” responses. Approaches to humor production often focus on the output, offering explanations in terms internal to the producer, such as creativity (Kozbelt, 2019), expertise and intelligence (Derks et al., 1998), or personality traits (Nusbaum et al., 2017). Social context is acknowledged, but rarely theorized. Concepts such as “joking cultures” (Fine & De Soucey, 2005) and “humor communities” (Carrell, 1997), however, imply the point that social context is not solely based on the fact that humor is enacted with others, but that these others are often valued groups, and that being a member of these groups means being an active participant in humor exchanges. Thus, through individuals’ desires to belong, group norms shape the strategic and rhetorical uses of humor.

One approach that focuses on social norms is the theory of normative social behavior (TNSB; Lapinski & Rimal, 2005; Rimal & Real, 2005). The theory emphasizes that we often act in social situations in ways that are similar to those around us, especially when we perceive those others to be like us. To date, TNSB has been used to examine a number of health-related behaviors, with potentially deleterious outcomes, including drinking (Real & Rimal, 2007), prescription medicine misuse (Jain & Humienny, 2020), food consumption (Varava, 2019), cheating (Henningsen & Henningsen, 2020), and handwashing (Lapinski et al., 2014). However, there is nothing in the theory that restricts its application to harmful or dangerous behaviors. All socially enacted behaviors, including humor, are shaped by relevant group norms. This investigation uses TNSB to explore the influence of norms on the uses of humor. Additionally, acknowledging that humor is multifaceted and can be used for diverse purposes, this study adopts J. C. Meyer’s (2000) four functions of humor approach.

## 2. Theory of Normative Social Behavior

The core feature of TNSB is the relationship between descriptive norms and the target behavior (Lapinski & Rimal, 2005). Descriptive norms involve perceptions of what referent group members actually do, i.e., what is normative in a given situation. These perceptions are developed through observation and communication. Though research on the everyday uses of humor is scarce, it supports the oft-repeated assertion that humor is a common activity in interpersonal settings, and that such humor is sensitive to the daily context of people’s lives (Nezlek et al., 2021). Using a diary method, Mannell and McMahon (1982) found that college students reported around 18 humor incidents a day, with the majority involving spontaneous interpersonal humor and the recounting of previous events. Beck et al. (2007) found that self-reported teasing among college students was a fairly frequent activity. The most commonly reported reasons for teasing were for fun, followed by bonding, cheering others up, and showing liking. In a diary study of romantic couples (J. Meyer, 2012), around 11 relationship-related incidents of humor per day were recorded. The events were overwhelmingly positive and enhanced the couple’s relationships. From the evidence presented, it can be inferred that a positively valenced activity, frequently enacted, would be both observable and something about which people communicate. Therefore, humor use should be affected by descriptive norms. However, the theory further argues that the influence of such norms is often conditional on other factors. In this study, we focus upon injunctive norms, group identification, outcome expectations, and the rewards of using humor itself.

Injunctive norms are prescriptive rules or expectations providing guidance on what one ought to do. Coupled with this sense of oughtness are perceptions of consequences, benefits that accrue for compliance, and/or sanctions that follow for noncompliance (Lapinski et al., 2014). In studies of humor, there appears to be little research directly bearing on such norms. One reason for this may be the association between humor and play. Contrasted with the serious world of work, the nonserious mode of play is supposed to be a realm of liberation and freedom (e.g., Mulkay, 1988). As such, the notion of oughtness implied in injunctive norms is antithetical to engaging humor. Conceptually, however, there is no necessary connection between freedom and the work/play distinction. Participation in serious endeavors can be liberating, and humor can be enacted with a serious purpose. Along similar lines, lacking a sense of humor is associated with being dour, inflexible, authoritarian, even boring (Wickberg, 1998). Everyone supposedly knows that they ought to have a sense of humor, being able to laugh at themselves and appreciate the humor of others; it can thus be inferred that a sense of oughtness makes some contribution to the everyday ways people enact humor.

Group identification refers to the referent group for the behavior in question. According to Rimal and Real (2005), it includes “perceptions of similarity and desire for connection with [a] reference group”. Though it is often claimed that humor can promote perceived similarity and common ground, this is usually with one’s immediate interlocutor, and not a broader group with whom one identifies. On the other hand, a long tradition of research suggests that joke preferences are affected by group membership (La Fave et al., 1996; Zillmann & Cantor, 1996). In this study, the reference group is college students, and there are some grounds for supposing that college students are exposed to a great deal of humor, including political humor, intragroup teasing, and shared memes (Penney, 2020). It is posited that students use humor to enact or reflect the sense of similarity and identification felt toward members of the referent group.

Outcome expectations involve beliefs about the consequences of performing a behavior, especially focused upon desired results (Lapinski et al., 2007). Numerous studies attest to the positive outcomes of using humor. In a study of humor and mate selection, McGee and Shevlin (2008) found that message targets described as possessing a good sense of humor were rated as more attractive and more suitable as a mate than those described as being average or having no sense of humor. In an organizational setting, Rizzo et al. (1999) found that managers rated higher in Humor Orientation (HO; Booth-Butterfield & Booth-Butterfield, 1991) were perceived as more likable and more effective. A study of classroom communication found that teacher HO was positively related to self-reported student learning (Wanzer & Frymier, 1999). Humor is also promoted for its many health or coping benefits (Martin, 2004). Given that we are surrounded by discourses of humor, as well as personal experience and observation, it is expected that using humor is associated with positive outcomes.

The variables described above form a commonly used set of moderators. TNSB is flexible in admitting additional factors, including ego involvement (Lapinski et al., 2007), peer communication (Real & Rimal, 2007), and publicness (Lapinski et al., 2014). In this investigation, the focus is on a form of communication rather than a particular health-related behavior. Therefore, finding humor a rewarding and enjoyable activity was considered a potentially relevant moderator to include along with the more traditional ones (i.e., norms and outcome expectations). Several measures of humor use refer to how often or how frequently participants use a particular form of humor. Given that people might use humor for various purposes, the pleasure derived from telling funny stories or joking around is considered separable from those overarching motives. For instance, a person might enjoy enacting humor but have few opportunities to do so, while another might not find it pleasant even though they use it for particular social purposes. It is assumed that, most often, those who enjoy the activity of humor itself use it in more and varied ways. As such, reward is included as a potential moderator of the descriptive norm–humor use relationship.

To this point, humor has been presented as an undifferentiated construct. It is widely recognized, however, that humor can take different forms (Cann et al., 2009). Given the present emphasis on how social norms shape the strategic uses of humor, J. C. Meyer’s (2000) functional approach was adopted.

## 3. Functions of Humor

Humor helps people navigate through and share many elements of social and organizational life. Social norms can be elicited, reinforced, avoided, or even defied by invoking humor. Indeed, “[h]umor use seems to provide morally, politically, or relationally safe ways to express ideas in spite of the restrictions that any kind of order—even an order voluntarily produced or reproduced—will inexorably impose” (J. C. Meyer, 2015, p. 26). Humor’s key social functions can be grouped on a continuum from most unifying to most divisive, constituting the identification, clarification, enforcement, and differentiation functions.

### 3.1. Identification Humor

Identification humor allows for a sense of belonging, as participants share a common perception or value that is elicited and shared in some way, even if the source of humor violates social expectations. The mutual understanding of how a certain pattern or norm is socially dominant reinforces group or dyad cohesion and solidarity. Identifying with others through humor is the most positive or pleasant function of humor. We like sharing some of the same expectations and understandings of our shared social world. The sense of commonality serves as a strong social connector, or glue providing a solid basis for further interactions.

Identification humor can construct group cohesiveness (Graham et al., 1992) as well as reduce uncertainty about others by appealing to common values (J. C. Meyer, 1997, 2015). The dominant expectation or norm is emphasized by this function of humor, and a solid shared perspective is evoked. An instance of some unusual violation of an expected pattern sparks the humor experience, but the mutual reinforcement of that shared pattern is dominant for identification. A common understanding of events is crucial for such humor and results in a strengthening or reinforcement of the relationship.

### 3.2. Clarification Humor

Clarification humor highlights a contrast or difference in a new way—the shared sense of understanding underlies a twist or unusual perception or phrasing incorporating it. A funny joke or phrase where one gets a sense of saying “that’s so true” provides a good example of the clarification function (J. C. Meyer, 2000). One can memorably drive home a point on an issue through a humorous remark. The strong shared norm or expectation is combined with the violation sparking the humor to advance knowledge or understanding of the topic farther along, reinforcing a shared norm with a new perspective added to it.

Clarifying as a function of humor provides “gotcha” moments where we understand a norm or expectation in a new way, often through a new or unique violation of it. A new path of thinking or perceiving is enabled even as the underlying expectation or norm is reinforced for the perception of humor. “People enhance relationships through laughing together at such remarks that effectively reinforce social norms—by showing that the background norms and expectations still endure” (J. C. Meyer, 2015, p. 36). Funny remarks, with ideas expressing and reinforcing norms in a way not thought of before, provide a sense of unity and strengthen shared norms and expected patterns. People participating feel more confident and have their uncertainty about social or physical expectations reduced as a result, making the humor experience pleasant and unifying in general.

### 3.3. Enforcement Humor

Enforcement humor emerges from reinforcing a norm or expectation where one stresses a violation as noteworthy and potentially laughable. A breaking of the norm or violation of the expectation results in humor impressing on participants that a correction or improvement may be necessary. Duncan (1962) pointed out that humor can enforce social norms through a kind of “discipline by laughter”. The violation of norms or expectations is, in these instances, highlighted somehow as more dramatic or important, worthy of laughter in part because the violation should not continue. People may then avoid similar additional violations of norms or expectations to avoid being laughed at.

Enforcement humor is essential to teasing—we build or reinforce relationships through teasing that emphasizes mutual expectations that should be followed, and, if not, we will give one another a “hard time” about them. Teases can serve a corrective function while maintaining some positive relationship through implied concern for the other party (Young & Bippus, 2001). They can also strain and stress relationships—the norms that are held as dominant may be difficult or unjustified in the eyes of the other, threatening a person’s mental state or sense of belonging in the social relationship. There is a sense in teasing of “conform, or else …” We may often ponder: how much of teasing is merely the fun pointing out of a certain uniqueness or difference, and how much is implied or real criticism that may require action? “Enforcement, in the end, has an element of aggression or criticism, which may or may not be mitigated by reinforcement of a strong relationship or cohesive group” (J. C. Meyer, 2015, p. 39). An undesired challenge by or refusal to face the consequences of teasing may damage the relationship, and conversely, facing and responding to teasing may show an ability for a relationship to stand up to challenges and stresses.

### 3.4. Differentiation Humor

Differentiation humor involves contrasts—one group from another, or one perspective against another. The dramatic differences between the socially expected norm or pattern and its violation that sparked the humor are highlighted. This humor function reveals social alliances and divisions. The comfortable “familiar” is contrasted with a wayward or disturbing strange phrase or event. People laugh together at humorous accounts of an “other” that is viewed as ignorant or wrong. The violation or clash with the expected normative pattern that leads to humor appreciation is stressed. This humor function emphasizes the difference noted as contrasted with the expected or familiar (J. C. Meyer, 2000). Thus, whereas clarification humor provides “optimal distinctiveness” (Brewer, 2003) within the ingroup, differentiation humor emphasizes the distinction of ingroup members from some outgroup.

People understand the dreaded feeling resulting from a sense of being laughed at, which implies becoming an object of ridicule, or in some way no longer accepted by those laughing. Such drawing of social distinctions can be cruel yet clear—those “in” laugh along with others, but those “out” get laughed at as the alienated other. Humor use can thus differentiate between all sorts of social groupings. One who is differentiated out may “get” the joke, but also be ego-involved in the topic and feel a threat to one’s own identity (J. C. Meyer, 2015) and thus not share in the humor. Or, one may not “get” the joke at all, since the pattern being violated is not perceived or understood, or viewed as not worthy of notice. This may make being laughed at even more irritating, in a way, since one is not sure why. Laughing with the “in” group may be rewarding and even serve paradoxically as identification or unifying humor, but to one who feels “out”, such humor may feel socially alienating indeed.

### 3.5. Humor in Social Groups

In society, the key humor functions play out in many ways crucial to social life. Humor can unify groups who work on and perceive tasks from similar perspectives. The shared expectations reinforce group solidarity and opinion. Identification humor stresses those similarities and understandings. Clarification humor can serve to revive and reify useful shared perspectives with new energy, as perspectives are appreciated in a new and unique way, varying enough from expectations to spark humor appreciation, but reinforcing understood norms to strengthen a work group’s cohesion. Humor use has been found essential as part of worker motivation in organizations—more humor use has often been associated with a positive emotional climate and work and effort in teams (Lutgen-Sandvik et al., 2011). People who can laugh together have a sense of safety (Miczo et al., 2009) to be creative and work more closely together. Humor serves a variety of unifying functions for work teams according to research, including managing group behaviors while avoiding conflicts and maintaining group cohesiveness, increasing solidarity, and constructing collective identity among members (Moalla & Amor, 2021).

Humor certainly enhances group cohesion; yet, it also reinforces group hierarchy, by reinforcing and correcting for social norms (Ben-Ari & Sion, 2005; Lynch, 2009; Ziv, 2010). Enforcement humor in a social group has an “edge” to it—a noteworthy violation of expectations is mocked and made light of, stressing the violation rather than the ongoing norm. With jokes about norm or pattern violations, those very norms and patterns are reinforced as important and ones that matter, as violations of them are viewed as noteworthy. After all, “laughing at those who violate norms motivates all to conform to them” (J. C. Meyer, 2015, p. 73). People will often conform in groups largely to avoid being made light of or mocked through differentiating humor, thus reinforcing organizational norms.

Humor is also essential to new social group members becoming assimilated, as many norms and expectations can be learned through joking about violations, or through humorous stories of the same (Heiss & Carmack, 2012). Overall, humor may be more pleasant for learning about social expectations and norms and one’s own place within them. “Invoking and learning desired cultural patterns can be done with flexibility and forgiveness through humor, rather than through a more formal error-correcting form of communication” (J. C. Meyer, 2015, p. 77).

Norms and values are at the heart of what keeps social groups going, and humor is a natural output of noticing contrasts, violations, changes, or evolutions of them. Necessary for appreciating humor is a sense of an expected norm, along with its violation, in the mind at the same time (J. C. Meyer, 2000; Veatch, 1998). Thus, humor is a key way of dealing with cultural norms in organizations, whether reinforcing them or seeking to change them (J. C. Meyer, 2015). “Humor reframes potentially divisive events into merely ‘laughable’ ones which are put in perspective as subservient to unifying values held by organization members” (J. C. Meyer, 2015, p. 72). Sharing communication involving humor creates or enhances ties among group members (Holmes & Marra, 2002; Korczynski, 2011).

Through enactments of the four key humor functions, groups and relationships are formed and reinforced. Thus, social norms will impact the humor functions invoked. People seek to “fit in” and, thus, will engage in humor with a preference for socially rewarded functions, while avoiding social sanctions impending for norm-violating functions. A desire for a positive, creative environment, for instance, may lead to social norms favoring unifying, “safe” identification or clarification humor, while a highly competitive or “edgy” social group might encourage enforcement or even differentiating humor, so that members must prove that they can “take it” in given instances. The interaction between social norms and humor functions that emerge is worth further study.

## 4. Hypotheses and Research Questions

The premise of our argument is that, as a social activity, humor use is influenced by social norms. According to TNSB (Lapinski & Rimal, 2005; Rimal & Real, 2005), descriptive norms are a foundational influence, as people behave in line with their perceptions of what others are doing, including humor use. Insofar as humor can be used in different ways, it is not clear if J. C. Meyer’s (2000) four functions of humor will be differentially responsive to the impact of descriptive norms. The first hypothesis and research question reflect this basic premise:

**H1:**  *Descriptive norms significantly predict the four functions of humor (identification, clarification, enforcement, differentiation).*

RQ1: What is the relationship between descriptive norms and each of the four functions of humor?

TNSB (Lapinski & Rimal, 2005; Rimal & Real, 2005) proposes that descriptive norms may be particularly salient in the presence of relevant moderators. Thus, to the extent that one perceives others are using humor, and one feels one should be producing humor (i.e., injunctive norms), identifying with humor-using peers, expecting positive outcomes from engaging humor, and finding it a rewarding activity, one may be especially likely to produce humor. Once again, however, it is not clear if these interaction effects will hold across the four types of humor in J. C. Meyer’s (2000) model. This line of reasoning leads to the second hypothesis and research question:

**H2:**  *Relationships between descriptive norms and the functions of humor will be stronger at higher levels of (a) injunctive norms, (b) group identification, (c) outcome expectations, and (d) reward.*

RQ2: Will proposed interactions between descriptive norms and the moderating influences (a) injunctive norms, (b) group identification, (c) outcome expectations, and (d) reward differ across the four functions of humor?

## 5. Method

### 5.1. Participants

Participants (*n* = 163) were a nonrandom sample of students at a medium-sized university from the Midwestern U.S. The sample had an average age of 26.15 (*SD* = 10.11, range 18–68), with 99 females (60.7%), 59 males (36.2%), 2 non-binary/third gender (1.2%), and 3 preferring not to answer (1.8%). Ethnic composition was 110 Caucasian/White (67.5%), 29 Black/African American (17.8%), 10 Asian/Pacific-Islander (6.1%), 2 American Indian/Alaskan Native (1.2%), and 12 Other (7.4%). The sample was evenly spread across collegiate level: 21 freshman (12.9%), 30 sophomore (18.4%), 37 junior (22.7%), 48 senior (29.4%), and 27 graduate (16.6%).

### 5.2. Procedure

Upon approval from the university’s Institutional Review Board, an online survey was constructed using Qualtrics and posted to a participant research pool in the Department of Communication. The first section of the survey contained items related to the TNSB. The second section contained a measure of humor functions. The final section consisted of demographic questions. The majority of students received extra credit for their participation.

### 5.3. Measures

Items for descriptive norms, injunctive norms, outcome expectations, and reward were drawn from prior research on TNSB (cf., Lapinski et al., 2014; Rimal & Real, 2005). A preliminary series of confirmatory factor analyses were conducted using Jamovi 2.6 (The jamovi project (2024). jamovi (Version 2.6) [Computer Software]. Retrieved from https://www.jamovi.org). Those analyses revealed that the model fit was poor for injunctive norms, outcome expectations, and reward. Accordingly, an exploratory factor analysis was conducted on the four subscales. The EFA, using principal axis factoring with oblique rotation, yielded three factors, accounting for 51.8% of the variance; Bartlett’s χ^2^ (120) = 1260, *p* < 0.001; KMO = 0.84. The three variables described below are based on that EFA; the Age Identity Scale was considered a separate measure of group identity and treated accordingly.

Descriptive norms. Descriptive norms were measured with four items adapted from Lapinski et al. (2014), who refined them into a reliable instrument (e.g., “Most peers on campus engage in joking around with one another”) (19.9% of the variance in the EFA). Lapinski et al. (2014) state that they derived their items from prior research on TNSB. However, their subscales were designed to be short and, through CFA, were shown to be unidimensional. They have also been used in subsequent research (e.g., Lapinski et al., 2017). As such, they were deemed to provide suitable models for present purposes. Items were assessed with 5-point Likert-type scales (1 = “Strongly Disagree” and 5 = “Strongly Agree”). The alpha reliability for the scale was 0.87 (*M* = 3.75, *SD* = 0.81). A CFA produced a reasonable fit: χ^2^ (2) = 1.28, *p* = 0.52; CFI = 1.00; RMSEA = 0.00 (0.00, 0.14).

Injunctive norms. Two items from the original injunctive norms measure (“Peers may judge me based on whether or not I tell funny stories”, “I feel like peers would think less of me if I didn’t joke around”) and two items from the original outcome expectations measure (“I will be less likely to be alone if I joke around with my peers”, “I will be more popular if I make my peers laugh”) formed the second factor (16.1% of the variance). This factor was named injunctive norms insofar as it captured the social motivations arising from a sense of how one ought to be among one’s peers. Items were assessed with 5-point Likert-type scales (1 = “Strongly Disagree” and 5 = “Strongly Agree”). The scale exhibited adequate reliability (α = 0.76; *M* = 3.26, *SD* = 0.84). A CFA produced a reasonable fit: χ^2^ (2) = 1.41, *p* = 0.50; CFI = 1.00; RMSEA = 0.00 (0.00, 0.14).

Reward. The third factor (15.8% of the variance) included four items concerning positive attitudes toward the activity of joking (“Joking around with peers is enjoyable”, “Joking around with peers is rewarding”, “Joking around with peers is pleasurable”, “Joking around with peers is fun”) adapted from Rimal and Real (2005). Items were assessed with 5-point Likert-type scales (1 = “Strongly Disagree” and 5 = “Strongly Agree”). The alpha reliability for the scale was acceptable (α = 0.80; *M* = 4.28, *SD* = 0.69). A CFA produced an acceptable fit: χ^2^ (2) = 21.6, *p* < 0.001; CFI = 0.92; RMSEA = 0.25 (0.16, 0.34).

Group identity. The propensity to identify with one’s age group was measured with the 13-item Age Identity Scale (AIS; McCann et al., 2004) (e.g., “I like being a member of my age group”). Items were assessed with 5-point Likert-type scales (1 = “Strongly Disagree” and 5 = “Strongly Agree”). The alpha reliability for the scale was 0.93 (*M* = 3.52, *SD* = 0.80). A CFA produced an acceptable fit: χ^2^ (65) = 171, *p* < 0.001; CFI = 0.91; RMSEA = 0.10 (0.08, 0.12).

Functions of humor. Ramsey & Meyer’s (2019) 19-item functions of humor scale was utilized to assess participants’ humor usage. All items were assessed with 5-point Likert-type scales (1 = “Strongly Disagree” and 5 = “Strongly Agree”). Identification humor (e.g., “Using humor helps me feel closer to others”) was measured with 5 items (α = 0.85; *M* = 3.99, *SD* = 0.75). A CFA produced an acceptable fit: χ^2^ (5) = 3.27, *p* = 0.66; CFI = 1.00; RMSEA = 0.00 (0.00, 0.09). Clarification humor (e.g., “I use humor to get my point across”) was assessed with 5 items (α = 0.77; *M* = 3.69, *SD* = 0.78). A CFA produced an acceptable fit: χ^2^ (5) = 6.31, *p* = 0.28; CFI = 0.99; RMSEA = 0.04 (0.00, 0.12). Enforcement humor (e.g., “When someone makes a mistake, I often use humor to let them know”) was assessed with 5 items (α = 0.82; *M* = 3.12, *SD* = 0.89). A CFA produced an acceptable fit: χ^2^ (5) = 5.24, *p* = 0.39; CFI = 0.99; RMSEA = 0.02 (0.00, 0.11). Differentiation humor (e.g., “I use humor to assert my independence”) was measured with 4 items (α = 0.68; *M* = 3.58, *SD* = 0.86). A CFA produced an acceptable fit: χ^2^ (5) = 5.95, *p* = 0.05; CFI = 0.97; RMSEA = 0.11 (0.00, 0.22).

## 6. Results

H1 and RQ1 concerned the relationship between descriptive norms and the four functions of humor. H1 predicted that descriptive norms are related to the function of humor, while RQ1 concerned the particular pattern of the relationship with each function. As can be seen in Table 1, descriptive norms positively correlated with all four of the humor functions. In order to isolate the impact of descriptive norms on humor functions, as well as explore the unconditional effects, separate regression analyses were performed. Results of those analyses are depicted in Table 2. For identification, the overall model was significant, *F* (4, 158) = 48.66, *p* < 0.001, *R*^2^ = 0.55. Both injunctive norms (ß = 0.44, *p* < 0.001) and reward (ß = 0.49, *p* < 0.001) were significant predictors. For clarification, the overall model was significant, *F* (4, 158) = 10.35, *p* < 0.001, *R*^2^ = 0.21. Both injunctive norms (ß = 0.30, *p* < 0.001) and reward (ß = 0.31, *p* < 0.001) were significant predictors. For enforcement, the overall model was significant, *F* (4, 158) = 9.34, *p* < 0.001, *R*^2^ = 0.19. Only injunctive norms (ß = 0.40, *p* < 0.001) was a significant predictor. For differentiation, the overall model was significant, *F* (4, 158) = 14.18, *p* < 0.001, *R*^2^ = 0.26. Only injunctive norms (ß = 0.49, *p* < 0.001) was a significant predictor. Contrary to the first hypothesis, descriptive norms did not significantly predict any of the functions of humor. Rather, the most consistent and significant predictor of all four humor functions was injunctive norms. Thus, H1 was not supported, suggesting, in response to RQ1, no statistically meaningful pattern of relationships between descriptive norms and the functions of humor.

H2 and RQ2 focused upon the impact of the moderators (injunctive norms, group identification, outcome expectations, and reward) on the relationship between descriptive norms and humor functions. To assess these relationships, Hayes’s (2018) PROCESS Macro for SPSS (Model 1) was utilized. Table 3 and Table 4 present the moderation analyses for the functions of humor. Significant interactions were further probed, using PROCESS, by examining the effect of the moderators at the 16th, 50th, and 84th percentile. Those results are presented in Table 5.

**Figure 1:**
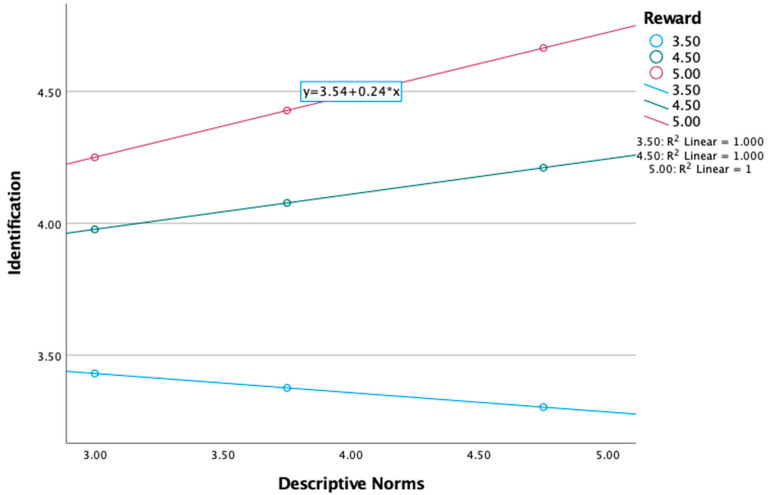
The interaction of descriptive norms and reward on identification.

**Figure 2:**
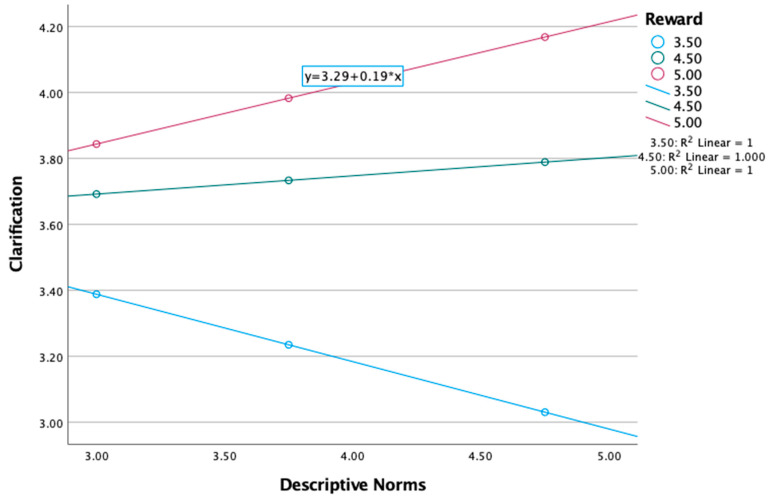
The interaction of descriptive norms and reward on clarification.

For identification humor, there was a significant interaction effect for reward, *F* (3, 159) = 39.43, *p* < 0.001, B = 0.20, *p* < 0.01. As can be seen in Table 5, the effect of reward on descriptive norms was significant at the 84th percentile, B = 0.22, se = 0.08, *p* < 0.01 (0.06, 0.38). Thus, the influence of descriptive norms on identification was conditional on higher levels of reward. See Figure 1. The pattern depicted in Figure 1 is similar to the descriptive norms × reward for enforcement and descriptive norms × reward for differentiation interactions reported in Table 5. The only exception is the descriptive norms × reward effect on clarification depicted in Figure 2. Therefore, only Figure 1 is provided here.

For clarification humor, there was a significant interaction effect for reward (see Table 5), *F* (3, 159) = 11.97, *p* < 0.001; B = 0.26, *p* < 0.01. Regarding reward, the significant interaction was negative at the 16th percentile, B = −0.21, se = 0.10, *p* < 0.05 (−0.41, −0.00). Thus, at lower levels of reward, descriptive norms had an impact on clarification humor. See Figure 2.

For enforcement humor, there was a significant interaction effect for reward, *F* (3, 159) = 7.21, *p* < 0.001; B = 0.31, *p* < 0.01. As can be seen in Table 5, the effect of reward on descriptive norms was significant at the 84th percentile, B = 0.33, se = 0.12, *p* < 0.01 (0.09, 0.56). Thus, the influence of descriptive norms on enforcement was conditional on higher levels of reward.

For differentiation humor, there was a significant interact effect for reward (see Table 5), *F* (3, 159) = 8.02, *p* < 0.001; B = 0.29, *p* < 0.01. For reward, the interaction was significant at the 84th percentile, B = 0.34, se = 0.11, *p* < 0.01 (0.13, 0.56). Thus, the influence of descriptive norms on differentiation humor was conditional on higher levels of reward.

Overall, these results offer partial support for H2. That is, of the twelve moderation analyses, four of the interactions were statistically significant. Regarding RQ2, reward had the most consistent impact on the relationships between descriptive norms and humor functions.

## 7. Discussion

This investigation examined the functions of humor using the theory of normative social behavior (Lapinski & Rimal, 2005; Rimal & Real, 2005). Descriptive norms, or perceptions of the ways others behave, are the foundation of TNSB. However, the theory argues that the impact of those norms is influenced by a set of moderators that can strengthen or attenuate proposed relationships. Though descriptive norms significantly correlated with all four humor functions, in regression analyses, no significant associations emerged. On the other hand, injunctive norms consistently predicted humor functions. This suggests people engage in humor because they feel they ought to do so. People know that they should try to be funny, or at least find ways to appreciate humor, in the context of their social groups. Once those pressures develop, members find they no longer are entirely free with their performances. Injunctive norms, as guides to behavior in social groups, may function to pressure persons toward invoking humor. In moderation analyses, the interaction between descriptive norms and reward was significant for all four functions of humor. This suggests that observations of how others use humor do, in fact, provide a foundation for one’s own humor efforts, as long as one enjoys engaging in humor.

### 7.1. Descriptive Norms and Humor Functions

Humor is a valued trait (Wickberg, 1998), and, therefore, to be humorous is to lay claim to a socially valuable attribute, and to enact a positive identity. Such an identity is grounded in our observations of, and communication about, what those around us do in their interactional lives. Descriptive norms may act like data, accumulated during our lives, providing observations of what others in our social group do. Insofar as groups are more than aggregates of individuals, “joking cultures” (Fine & De Soucey, 2005) develop and prescriptive norms emerge. These injunctive norms are guides or commands that tell us what we ought to do. Such social mandates push group members to take social risks and use some creativity to enact humor. Social pressure seems to encourage enacting the functions of humor in social groups. Identification and clarifications functions serve to strengthen social groups with added unity. Enforcement and differentiation functions reinforce norms and unique group characteristics differentiating one group from others. The influence of injunctive norms on humor functions may reflect the pressure to reinforce and distinguish social ties.

Looking across the four functions of humor, all four humor functions were predicted by injunctive norms. This suggests that when people feel pressured to create humor, they may use a variety of forms. The divisive functions of humor were only predicted by injunctive norms. Such humor use may seek unity of some sort in the face of social divisions or violations. The social group’s survival or reinforcement can be enhanced through humorous responses to differences or perceived social norm violations. The pattern was somewhat broader regarding the unifying forms of humor. That is, in addition to injunctive norms, identification and clarification humor were predicted by reward. With respect to the prosocial unifying functions, people use these forms of humor not just prescriptively, but also because they enjoy them. Creating positive social interactions that draw upon and reinforce common ground can be an end in itself.

Many forms of everyday humor, such as playful teasing, sarcastic comments, and joking around, occur among intimates, those known to one another with an established relationship of some sort. These interactions, however, remain embedded in a normative social order. The quality of the relationship is, to a large degree, dependent upon the efforts of partners to maintain it in a desired state. But social life is constantly disrupted by the flaws of everyday living—a partner who makes a thoughtless remark, farts in church, or petty acts of disruption. To never address these minor gaffes and faux pas can become a message in its own right. Humor provides an alternative means of raising the issue of a social violation. Identification humor conveys that the partner is loved and accepted through the minor transgressions. Clarification humor, displaying the wit and verbal agility of the humorist, can be an attempt to enhance the relationship by making the humorist more attractive, more likable even in the act of poking fun at the partner. Thus, enjoyment of using interpersonal humor is grounded in the enjoyment of the relationship itself.

Humor is often viewed as an enjoyable phenomenon, facilitating social interaction and even criticisms. It is possible that, if these interactions become too pleasant, the point of the humor risks being lost. Humor may be fun, and that enjoyment may erase the social critique or correction inherent in divisive humor, or even the message of an important social norm grabbing attention through a violation sparking humor. This may explain why reward only predicted the unifying forms of humor (identification and clarification). A message of true criticism or warning may be lost in the enjoyment of humor, or humorous remarks reinforcing unity and social norms may divert attention from needed serious discussion of issues. Humor may function more as a social facilitator or marker more than as a persuader or social deliberator.

The divisive forms of humor (enforcement and differentiation) emphasize the violation of the expected state of affairs even at the expense of the partner. In this sense, disparagement humor is not about enhancing one’s relationship; rather, the key to such humor is the relationship between the humorist and a moral-normative social order that has been violated in some way. With enforcement humor, the focus is upon the behavior as a violation that ought to be corrected or remedied. More often than not, the target remains within the fold, not seriously at risk for exclusion, but not living up to some standard of the group. Use of humor to enforce norms has been noted by researchers. Lynch (2009), for example, discussed how professional chefs used humor to maintain standards of professionalism in the kitchen. Enforcement humor may take the form of teasing, but will the teasing be taken “in fun” or as serious criticism? The norms of the relationship and social group may determine that teasing is expected, even when some individuals may find teasing is “taken too far” as they become ego-involved in the issue and respond as if it were seriously intended criticism. However, the norms of the group may demand that one accept or go along with a humorous tease, rather than responding seriously and becoming a “stick in the mud”. The group’s norms are subtly reinforced by such teasing.

When differentiation humor is utilized, the target is sacrificed more completely to the moral order. The humor is directed at an “other”, presumably one not to be emulated. That is, the basis of exclusion is membership in a group, with the group itself falling short in some way compared to the particular moral order from which the humorist operates. For example, many jokes turn upon the “stupidity” of outgroups (Davies, 1998) or otherwise position the target as ridiculous in some way. Implicit within these incongruities is a social order with established norms of competence and appropriateness. Those who seek to share in such humor must acknowledge a violation, or a difference, that is in some way so unacceptable as deserving of laughter and ridicule. “They” are funny, and it is nice not to be “them”, doing what they do or say. Divisive humor may reinforce group or social norms in varied ways.

Of course, multiple functions of humor may be used simultaneously, as influenced by the given social norms of a situation. For instance, a clear putdown of an outgroup (differentiation function) could be made even as one believes one’s entire audience will take that putdown for granted as true, thus making the statement a funny clarification of one’s views in an imaginative or unique way. Like many rhetorical or conversational tools, humor functions may be invoked in “sets” at times, with multiple purposes at once. Future research should explore the communication or social orientations and differing goal states underlying the functions of humor. What situations or purposes underlay identification or clarification humor? What tends to lead to or promote more enforcement or differentiation humor? Motives may also play a part, as the end goal may cause more conscious choice of a humor function to enact.

### 7.2. Moderation Analyses

The results of the regression analyses must be tempered by consideration of the moderation analyses. In those results, reward, or the pleasure people derived from using humor, consistently interacted with descriptive norms in predicting functions of humor. The more participants perceived humor was something their peers did, and the more they found it a pleasant experience, the more they used humor in most of the ways outlined in Meyer’s model. The one exception was clarification humor, where the less rewarding they found humor use, the less they used this type of humor.

In line with TNSB’s expectation that descriptive norms are both observed and communicated about, people look around their social environments and see their peers using humor, and the effects of these interactions. Humor can be seen to bring benefits such as popularity and attractiveness. Humor, then, becomes an acceptable component of the “status game” (Storr, 2022) of social life. There are two routes to status, however: dominance and prestige (Cheng, 2020). Establishing and maintaining dominance can be carried out humorously in relatively straightforward ways, such as disparaging jokes and pranks. Prestige, on the other hand, requires skill and competence, and clarification might be one way to do this insofar as it involves expressing oneself through clever insights and phrasings in ways that preserve social bonds and relationships. Whereas bonding through identification humor may also be accomplished through finding things to laugh about (J. Meyer, 2012), clarification humor may be used mostly by those who find verbal word play a rewarding activity.

One way individuals observe humor is through participating in humorous exchanges. In other words, descriptive norms are likely formed, in part, by the awareness and enjoyment of joking around and telling stories with others. Thus, possessing a sense of humor may involve laughing at and appreciating its inclusion in communication, or simply an awareness that humor is being attempted without enjoying it. An additional active step may be taken when one seeks to enact or produce humor, creating it as well as enjoying it. One need not produce humor to appreciate it or have a sense of humor, but doing so will enact one or more of the functions of humor. Future research could therefore explore the importance of humor functions for both the producers and recipients of humor. Although convenient to assume these would be the same in any given statement or conversation, senders and receivers of messages do not necessarily receive the same one. Seeking to measure humor functions for both initiators and recipients of messages could be valuable.

## 8. Limitations

There are limitations of the sample. Given the ubiquity of humor on the college campus, college students are an appropriate sample, but they are also a narrowly chosen group. Interpretations of humor norms likely differ markedly among non-college emerging adults, as well as older adults in diverse occupations. Regarding the measures, some of the scales had lower-than-desired reliabilities. As a starting point from which to explore the role of social norms on humor usage, the TNSB measures were adapted from existing TNSB research as a matter of convenience. Future research is needed to establish clearly differentiated measures of injunctive norms and outcome expectations. Additionally, Ramsey & Meyer’s (2019) scale is still new.

One particular limitation concerns the performance of the group identity measure. It is possible that group identification is simply not relevant to the study of humor. More likely, the measure of identity used here (age-group) is too nebulous and ill defined to be effective. That is, the relevant group of associates is the “humor community” in which one is embedded, likely made up mostly of friends or, at least, acquaintances.

## 9. Conclusions

It is not uncommon for positive and negative measures of humor to show moderate correlations. Those who use one type of humor may be more inclined to use multiple types. One of the principal ways of making people laugh is to make fun of things, including other people. Those inclined to use humor will likely use it to serve all four of the functions, both the prosocial and divisive varieties. Yet, individuals likely have a propensity for one or two of the functions of humor that may outweigh, to some extent, descriptive or injunctive norms operative in social situations. The diversity of social life is enhanced by people enacting the varied social functions of humor, and the results here show that injunctive norms, or perceived social pressure to engage in or appreciate humor, correlate more with humor functions used than do mere descriptive norms, or impressions of what is happening in the group or society.

## Figures and Tables

**Table 1:** Correlations among study variables.

	1	2	3	4	5	6	7	8
1. Descriptive norms	--							
2. Injunctive norms	0.40 **	--						
3. AIS	0.36 **	−0.01	--					
4. Reward	0.47 **	0.36 **	0.26 **	--				
5. Identification	0.36 **	0.59 **	0.12	0.63 **	--			
6. Clarification	0.17 *	0.36 **	0.10	0.37 **	0.54 **	--		
7. Enforcement	0.19 *	0.42 **	0.09	0.25 **	0.44 **	0.54 **		
8. Differentiation	0.22 *	0.51 **	0.04	0.26 **	0.50 **	0.53 **	0.46 **	--

NOTE. * *p* < 0.05; ** *p* < 0.01. AIS = Age Identity Scale.

**Table 2:** Regression analysis predicting humor functions from TNSB variables.

	Humor Function			
	Ident.	Clar.	Enf.	Diff.
	ß	ß	ß	ß
Predictor				
Desc.	−0.06	−0.12	−0.05	−0.02
Inj.	0.44 **	0.30 **	0.40 **	0.49 **
AIS	0.02	0.06	0.07	0.03
Rew.	0.49 **	0.31 **	0.12	0.09
F	48.66 **	10.35 **	9.34 **	14.18 **
R^2^	0.55	0.21	0.19	0.26

NOTE. df (5, 157). ** *p* < 0.01. AIS = Age Identity Scale.

**Table 3:** Moderation analyses of TNSB variables on identification and clarification functions of humor.

	Identification	Clarification
	Coeff. R^2^	Coeff. R^2^
Model 1: Injunctive Norms	0.37 **	0.15 **
Desc.	0.15	−0.40
Inj.	0.51 *	−0.21
Desc. × Inj.	−0.01	0.14
Model 2: Age Identity	0.13 **	0.03
Desc.	0.32	−0.01
AIS	−0.02	−0.13
Desc. × AIS	0.00	0.05
Model 4: Reward	0.43 **	0.18 **
Desc.	−0.79 *	−1.11 **
Rew.	−0.05	−0.47
Desc. × Rew.	0.20 **	0.26 **

NOTE. * *p* < 0.05; ** *p* < 0.01. AIS = Age Identity Scale.

**Table 4:** Moderation analyses of TNSB variables on enforcement and differentiation functions of humor.

	Enforcement	Differentiation
	Coeff. R^2^	Coeff. R^2^
Model 1: Injunctive Norms	0.18 **	0.26 **
Desc.	−0.18	−0.16
Inj.	0.17	0.25
Desc. × Inj.	0.07	0.06
Model 2: Age Identity	0.04	0.05
Desc.	0.19	0.09
AIS	−0.00	−0.20
Desc. × AIS	0.01	0.04
Model 3: Reward	0.12 **	0.13 **
Desc.	−1.2 **	−1.10 **
Rew.	−0.79 *	−0.77 *
Desc. × Rew.	0.31 **	0.29 **

NOTE. * *p* < 0.05; ** *p* < 0.01. AIS = Age Identity Scale.

**Table 5:** Interaction effects at 16th, 50th, and 84th percentiles.

	Percentile		
	16th	50th	84th
Identification Humor			
Desc. × Rew.	−0.08	0.12	0.22 **
Clarification Humor			
Desc. × Rew	−0.21 *	0.05	0.18
Enforcement Humor			
Desc. × Rew.	−0.13	0.17	0.33 **
Differentiation Humor			
Desc. × Rew.	−0.09	0.20 *	0.34 **

NOTE. * *p* < 0.05; ** *p* < 0.01.

## Data Availability

The dataset generated and/or analyzed during the current study are available from the corresponding author upon reasonable request.
